# 5-Hydroxy-7-Methoxyflavone Triggers Mitochondrial-Associated Cell Death via Reactive Oxygen Species Signaling in Human Colon Carcinoma Cells

**DOI:** 10.1371/journal.pone.0154525

**Published:** 2016-04-26

**Authors:** Monika Bhardwaj, Na-Hyung Kim, Souren Paul, Rekha Jakhar, Jaehong Han, Sun Chul Kang

**Affiliations:** 1 Department of Biotechnology, Daegu University, Kyoungsan, Kyoungbook 38453, Republic of Korea; 2 Department of Oriental Pharmacy, Wonkwang University, Iksan-city, Jeonbuk 570–749, Republic of Korea; 3 Metalloenzyme Research Group and Department of Integrative Plant Science, Chung-Ang University, Anseong 456–756, Republic of Korea; Faculty of Medicine & Health Sciences, UNITED ARAB EMIRATES

## Abstract

Plant-derived compounds are an important source of clinically useful anti-cancer agents. Chrysin, a biologically active flavone found in many plants, has limited usage for cancer chemotherapeutics due to its poor oral bioavailability. 5-Hydroxy-7-methoxyflavone (HMF), an active natural chrysin derivative found in various plant sources, is known to modulate several biological activities. However, the mechanism underlying HMF-induced apoptotic cell death in human colorectal carcinoma cells *in vitro* is still unknown. Herein, HMF was shown to be capable of inducing cytotoxicity in HCT-116 cells and induced cell death in a dose-dependent manner. Treatment of HCT-116 cells with HMF caused DNA damage and triggered mitochondrial membrane perturbation accompanied by Cyt c release, down-regulation of Bcl-2, activation of BID and Bax, and caspase-3-mediated apoptosis. These results show that ROS generation by HMF was the crucial mediator behind ER stress induction, resulting in intracellular Ca^2+^ release, JNK phosphorylation, and activation of the mitochondrial apoptosis pathway. Furthermore, time course study also reveals that HMF treatment leads to increase in mitochondrial and cytosolic ROS generation and decrease in antioxidant enzymes expression. Temporal upregulation of IRE1-α expression and JNK phosphorylation was noticed after HMF treatment. These results were further confirmed by pre-treatment with the ROS scavenger N-acetyl-l-cysteine (NAC), which completely reversed the effects of HMF treatment by preventing lipid peroxidation, followed by abolishment of JNK phosphorylation and attenuation of apoptogenic marker proteins. These results emphasize that ROS generation by HMF treatment regulates the mitochondrial-mediated apoptotic signaling pathway in HCT-116 cells, demonstrating HMF as a promising pro-oxidant therapeutic candidate for targeting colorectal cancer.

## Introduction

In recent decades, research methods for the discovery of natural compounds with potential anti-cancer activity have become streamlined. Flavonoids are naturally occurring polyphenolic metabolites found throughout the plant kingdom as well as in beverages such as tea and wine. Flavonoids are also non-essential dietary factors that provide an essential dietary link and helps prevent chronic diseases such as cancer. Anti-cancer activity exhibited by flavonoids depends upon their structure and composition as well as the type of cancer.

Colorectal cancer (CRC) is the second most common cancer malignancy [1], with an overall survival rate of only 5 years due to resistance to cytostatic drugs [2]. Major treatment options available for CRC include surgery alone or in combination with adjuvant chemotherapy, which is accompanied by radiotherapy or targeted therapy [3]. Current chemotherapeutic regimens for CRC are represented by fluoropyrimidine-based treatments such as 5-fluorouracil (5FU), cetuximab, panitumumab, paclitaxel, docetaxel, vincristine, oxaliplatin, and many others [4, 5].

Apoptosis (programmed cell death) has received much attention as a possible mechanism for the elimination of extensively proliferating cancerous cells. It is a highly ordered and orchestrated cell death mechanism involving activation of a series of molecular cascades. Apoptosis can be triggered through an extrinsic (death receptors) or intrinsic (mitochondrial) pathway. In the intrinsic pathway, mitochondria act as central integrators of apoptosis and are characterized by disruption of mitochondrial membrane potential, release of pro-apoptotic proteins into the cytosol (e.g. Cyt c, BID, Bax), subsequent caspase cascade activation, DNA fragmentation, chromatin condensation, and cell shrinkage [6]. Mitochondria are the prime source of reactive oxygen species (ROS), which are byproducts of mitochondrial aerobic respiration and play a crucial role in mitochondrial-mediated apoptosis. As mitochondrial signaling is altered in cancer cells, elevated ROS production is one of the outcomes of mitochondrial dysfunction. Mitochondrial dysfunction is one of the main therapeutic regimes among conventional therapeutic treatments, which are employed for targeting cancer cells [7]. As a result, there is an increased demand for anti-cancer drugs that elevate cellular ROS production from threshold levels in order to promote apoptosis in cancer cells. Besides altered mitochondrial metabolism, factors that contribute to up-regulation of pro-apoptotic factors and down-regulation of anti-apoptotic marker proteins are required to slow progression of cancer malignancies.

Activation of endoplasmic reticulum (ER) stress is one of the molecular mechanisms responsible for inducing signaling pathways that promote cancer cell death, thereby making ER stress a prominent target in cancer therapy. During ER stress, ER membrane-resident proteins, including PKR-like ER kinase (PERK), activating transcription factor 6 (ATF6), and inositol-requiring kinase 1 (IRE1), control a highly orchestrated signaling pathway known as the unfolded protein response (UPR), which inhibits or facilitates apoptosis [8, 9]. The outcome of cells towards survival or death depends upon the duration of stress. According to several earlier reports, ER stress-mediated apoptosis is triggered by IRE1-α-induced activation of JNK, which lies downstream of the IRE1 signaling pathway [10, 11]. Thus, activated JNK promotes phosphorylation of Bax by pathological activation of IRE1-α [12].

Earlier studies have demonstrated that natural compounds have the potential to trigger ROS generation, which leads to oxidative stress and perturbations in ER homeostasis. Chrysin (5,7-dihydroxyflavone), a bioflavonoid compound and proven antioxidant found in fruits and vegetables, is highly recommended for human consumption for cancer prevention [13]. The anti-cancer effects of chrysin are attributed to modifications in various signaling pathways that are involved in progression of cancer. Although the flavonol chrysin exhibits a wide spectrum of biological activities, its application for cancer therapy is limited since higher concentrations (>15 μg/ml) have been reported to be genotoxic in normal human-derived HepG2 cells [14]. Additionally, chrysin has low oral bioavailability in healthy humans due to extensive metabolism and metabolite efflux [15]. Therefore, derivatives of chrysin such as 5-hydroxy-7-methoxyflavone (HMF) could be suitable alternatives for cancer treatment.

There has been a tremendous increase in the demand for hydroxylated polymethoxyflavones (PMFs) due to their strong inhibitory properties against growth of human colon cancer cells [16]. To date, the mechanism of ROS-triggered cell death induced by HMF remains obscure. The present study was designed to investigate the potential anti-cancer effects of HMF, a derivative of ubiquitous flavonoid chrysin, isolated from *Kaemperia parviflora* of the Zingiberacea family against the human colon cancer cell line, HCT-116. In order to elucidate the mechanism of ROS-mediated mitochondrial apoptosis, we determined whether or not ROS act as mediators of HMF-induced cell death by activating molecular events leading to apoptosis. Further, our results show that the antioxidant NAC attenuated HMF-induced cell death by inhibiting ROS generation.

## Materials and Methods

### Reagents and antibodies

3-(4,5-dimethyl-2-thiazolyl)-2,5-diphenyl-2H-tetrazolium bromide (MTT), 2’,7’-dichlorodihydrofluorescein diacetate (H_2_DCFDA), Thiobarbituric acid (TBA), Hoechst 33342, Rhodamine-123 (Rh-123), Thapsigargin, Fura 2-AM and N-acetyl-l-cysteine (NAC) were purchased from Sigma (St. Louis, MO, USA). All antibodies used for Western blot analysis are listed in Table 1. All other chemicals and pure drugs were of reagent grade unless stated otherwise and were purchased from Sigma (St. Louis, MO, USA).

**Table 1:** List of Antibodies.

Antigen	WB dilutions	Source/Host	Catalog number	Company
β-actin	1:1000	Actin (I-19) Goat pAb	sc-1616	Santa Cruz Biotechnology
IRE1α	1:1000	IRE1α (H-190) Rabbit pAb	sc-20790	Santa Cruz Biotechnology
JNK	1:1000	JNK (FL) Rabbit pAb	sc-571	Santa Cruz Biotechnology
p-JNK	1:1000	p-JNK (Thr 183/Tyr 185) Goat pAb	sc-12882	Santa Cruz Biotechnology
Bcl2	1:1000	Bcl2 (N-19) Rabbit pAb	sc-492	Santa Cruz Biotechnology
BID	1:1000	BID (FL-195) Rabbit pAb	sc-11423	Santa Cruz Biotechnology
Caspase-3	1:1000	Caspase-3 (H-277) Rabbit pAb	sc-7148	Santa Cruz Biotechnology
Bax	1:1000	Bax (N-20) Rabbit pAb	sc-493	Santa Cruz Biotechnology
Cyt-c	1:1000	Cytochrome c (H-104) Rabbit pAb	sc-7159	Santa Cruz Biotechnology
Grp78	1:1000	Grp 78 (H-129) Rabbit pAb	sc-13968	Santa Cruz Biotechnology
PERK	1:1000	PERK (H-300) Rabbit pAb	sc-13073	Santa Cruz Biotechnology
eIF2α	1:1000	eIF2α (FL-315) Rabbit pAb	sc-11386	Santa Cruz Biotechnology
p-eIF2α	1:1000	p-eIF2α (Ser 52) Goat pAb	sc-12412	Santa Cruz Biotechnology
CHOP	1:1000	GADD 153 (R-20) Rabbit pAb	sc-793	Santa Cruz Biotechnology
Trx	1:1000	Trx (FL-105) Rabbit pAb	sc-20146	Santa Cruz Biotechnology
Prx	1:1000	Prx (N-19) Goat pAb	sc-7381	Santa Cruz Biotechnology
Glutathione reductase	1:1000	Glutathione reductase (H-120) Rabbit pAb	sc-32886	Santa Cruz Biotechnology
SOD-2	1:1000	SOD-2 (FL-222) Rabbit pAb	sc-30080	Santa Cruz Biotechnology
Rabbit IgG secondary antibody	1:10000	Goat anti-rabbit IgG-HRP	sc-2004	Santa Cruz Biotechnology
Goat IgG secondary antibody	1:5000	Donkey anti-goat IgG-HRP	sc-2020	Santa Cruz Biotechnology

### Compound isolation

5-Hydroxy-7-methoxyflavone (HMF) was isolated from *Kaemperia parviflora* by using the supercritical CO_2_ fluid extraction method. Optimal conditions of 200 bar of CO_2_ and 50°C were used for isolation of HMF, and structure was elucidated by UV-Vis and ^1^H NMR spectroscopic data analysis [17]. HMF was dissolved in 5% DMSO, and a concentration range of 25–100 μM was used for experimental analysis.

### Cell culture and treatments

HCT-116 cells, a human colorectal carcinoma cell line (ATCC, Rockville, MD), were maintained in a CO_2_ incubator at 37°C under a humidified atmosphere (95% air, 5% CO_2_) in RPMI 1640 medium supplemented with 10% fetal bovine serum, 25 mM HEPES buffer, and 1% Pen-Strep Cocktail (Gibco BRL, Gaithersburg, MD). Cells were treated with several concentrations of HMF (25, 50, and 100 μM) for 24 h to elucidate the underlying mechanism of apoptosis-induced cell death.

### MTT assay for cell survival

Cell survival was evaluated based on reduction of the water soluble yellow dye MTT into an insoluble blue formazan product. Cells were seeded at a density of 1x10^5^ cells in a 96-well flat-bottom microtiter plate and allowed to adhere for 48 h. After treatment with various concentrations of HMF for 24 h, 10 μl of MTT working solution (5 mg/ml in PBS) was added to each well for 4 h. The generated formazan crystals were solubilized by adding 50 μl of DMSO and quantified spectrophotometrically at 540 nm using an ELISA reader (Bio-Tek Instrument Co., WA, USA). The percentage of cell proliferation was calculated as (absorbance of treated cells/absorbance of control cells) x 100.

### Lactate dehydrogenase (LDH) assay for plasma membrane leakage

HMF-induced cellular cytotoxicity and cytolysis were determined using an LDH assay kit, which measures extracellular LDH in culture media using an enzymatic reaction to produce a red formazan product (Sigma–Aldrich, St. Louis, USA). LDH is a cytosolic enzyme that is released into the media by damaged cells as a biomarker for cellular toxicity. Briefly, HCT-116 cells (1 × 10^5^ cells/well) were seeded in a 96-well plate and treated with different concentrations of HMF for 24 h. After incubation, medium was collected and further procedures performed according to the manufacturer’s protocol. Cytotoxicity was quantified based on the amount of total LDH released by measuring absorbance of samples at 490 and 690 nm (Bio-Tek Instrument Co., WA, USA).

### Detection of nuclear condensation by Hoechst staining

After treatment, cells were washed twice with PBS and fixed with 4% formaldehyde for 30 min. The fixed cells were stained with Hoechst 33342 dye (1 μg/ml) for 15 min in the dark [18]. The stained cells were washed with PBS, and fluorescent micrographs were obtained using a Nikon Eclipse TS100 Epi-fluorescence microscope (Japan).

### Determination of intracellular ROS generation by H_2_DCFDA and mitochondrial superoxide level

H_2_DCFDA is a widely used compound for directly measuring the redox state of cells. H_2_DCFDA is deacetylated by esterases and then oxidized by ROS to form the highly fluorescent product 2’,7’-dichlorofluorescein [19]. HCT-116 cells treated with various concentrations of HMF for 24 h and 100 μM of HMF for 6–24 h before staining with 20 μM H_2_DCFDA for 15 min in the dark. Cells were then washed with PBS and visualized with a fluorescence microscope (Nikon Eclipse TS100 Epi-fluorescence microscope, Japan).

To examine the accumulation of mitochondrial superoxide, cells treated with various concentrations of HMF for 24 h and 100 μM of HMF for 6–24 h, were incubated with 5 μM MitoSOX Red mitochondrial superoxide indicator (Invitrogen) for 10 min, and then were washed twice with PBS. Fluorescent images were captured using a fluorescence microscope. Mean fluorescence intensity was quantified using ImageJ software after background staining correction.

### Thiobarbituric acid-reactive substances (TBARS) assay

Lipid peroxide content of HCT-116 cells was determined by using the modified TBARS assay method as described previously [20]. HCT-116 cells were treated with various concentrations of HMF for 24 h, after which the culture media were collected. An equal volume of 0.67% (w/v) TBA was added to the media, and the resulting supernatant was mixed with 100 μl of 0.1 M 2-TBA. The mixture was heated at 100°C for 10 min, after which the absorbance of TBARS was read at 532 nm (Bio-Tek Instrument Co., WA, USA). MDA standard was prepared from 1,1,3,3-tetraethoxypropane.

### Measurement of mitochondrial membrane potential (Δψm)

Alterations in mitochondrial membrane potential (MMP) were determined by using the fluorescent dye Rh-123 staining method as reported earlier [21, 22]. Briefly, cells were seeded in a 6-well plate and treated with different concentrations of HMF for 24 h. Cells were washed with PBS and stained with 1 μg/ml of Rh-123 at 37°C for 30 min in the dark. After washing with PBS, fluorescence intensity of cells was analyzed under a fluorescence microscope (Nikon Eclipse TS100 Epi-fluorescence microscope, Japan). Mean fluorescence intensity was quantified using ImageJ software after background staining correction.

### Intracellular calcium measurement

Fura 2-AM is a cell-permeable fluorescent probe that is generally used to measure ER Ca^2+^ levels. HCT-116 cells were seeded in a 6-well plate and then treated with various concentrations of HMF for 24 h and 2 μM of thapsigargin for 7 h. Thapsigargin was used as a positive control to induce intercellular Ca^2+^ level [23]. Cells were then loaded with 5 μM Fura 2-AM for 60 min at 37°C to allow for deesterification of Fura 2-AM. Cells were then washed three times and perfused with HEPES buffer, after which fluorescence intensity of cells was analyzed under a fluorescence microscope (Nikon Eclipse TS100 Epi-fluorescence microscope, Japan). Mean fluorescence intensity was quantified using ImageJ software after background staining correction.

### RNA isolation and quantitative real-time PCR analysis

Total RNA content of cells was isolated using a RNA-spin^™^ Total RNA extraction kit according to the manufacturer’s protocol (Intron Biotechnology, Korea). RNA concentrations were determined using a Qubit^®^ 2.0 Fluorometer RNA assay kit (Life Technologies, USA). cDNA was prepared using a Maxime RT Premix cDNA synthesis kit (Intron Biotechnology, Korea) according to the manufacturer's protocol. Quantitative PCR was performed using 0.9 μM each of forward and reverse primers (Table 2) for *bax*, *bcl-2*, *β-actin*, and *b2m* along with SYBR Green premix (Life technologies, USA) on a real-time PCR system (Agilent Technology QPCR System, CA, USA). RT-PCR control *β-actin* and *b2m* were amplified under the same PCR parameters to normalize quantitative data.

**Table 2:** Sequences of RT-PCR oligonucleotide primers specific for human *bax*, *bcl-2*, *β-actin* and *b2m*.

Gene	Direction	Sequence
*bax*	Forward	5’-CTG CAG AGG ATG ATT GCC G-3’
	Reverse	5’-TGC CAC TCG GAA AAA GAC CT-3’
*bcl-2*	Forward	5’-TCC CTC GCT GCA CAA ATA CTC-3’
	Reverse	5’-ACG ACC CGA TGG CCA TAG A-3’
*β-actin*	Forward	5’-AAC TAC CTT CAA CTC CAT CA-3’
	Reverse	5’-GAG CAA TGA TCT TGA TCT TCA-3’
*b2m*	Forward	5’-AGA TGA GTA TGC CTG CCG TG-3’
	Reverse	5’-TCA TCC AAT CCA AAT GCG GC-3’

### Assessment of Cytochrome c release from mitochondria

HCT-116 cells (1 × 10^5^ cells/ml) were incubated with 25, 50, and 100 μM HMF for 24 h or 10 mM H_2_O_2_ for 4 h. Cells were homogenized in buffer A (50 mM Tris, 2 mM EDTA, 1 mM phenylmethylsulfonyl fluoride, pH 7.5), after which 2% glucose was added to eliminate impurities and the mixture centrifuged at 2000xg for 10 min. Supernatants were gathered for measurement of released cytoplasmic Cyt c. Mitochondria were obtained by washing the pellet in buffer B (50 mM Tris, 2 mM EDTA, pH 5.0) by centrifugation at 5000xg for 30 s. Mitochondria were then suspended in 2 mg/ml of Tris–EDTA buffer. After reduction by 500 mg/ml of ascorbic acid for 5 min, Cyt c contents in mitochondrial samples were measured at 550 nm (Bio-Tek Instrument Co., WA, USA) [24].

### Mitochondrial protein isolation

Mitochondria-enriched fractions were prepared from HCT-116 cells grown to 90% confluency. Cells were pelleted by centrifugation and suspended in Mitochondrial Extraction Buffer (70 mM sucrose, 200 mM mannitol, 10 mM HEPES, 1 mM EGTA, pH adjusted to 7.5), followed by homogenization using a Dounce homogenizer. After cell disruption, homogenates were centrifuged at low speed (600×g). The resulting pellet was discarded, after which the supernatant was transferred into a fresh tube and centrifuged at high speed (11,000×g) to separate mitochondria from soluble cytosolic proteins. Protein concentration was defined using the Bradford method.

### Western blotting

Cells were exposed to various concentrations of HMF and lysed in RIPA buffer (Sigma–Aldrich, St. Louis, USA). Protein quantification was determined by using Bradford reagent, after which 50 μg of total proteins was separated and transferred to a PVDF membrane (Roche Diagnostics, USA). Membranes were subsequently immunoblotted with appropriate primary and secondary antibodies (Santa Cruz Biotechnology, Dallas, TX, USA), and signals were detected by enhanced chemiluminescence ECL system (Amersham, Velizy-Villacoublay, France).

### Statistical analysis

Each experimental condition was performed in triplicate (n = 3), and all data were expressed as the mean ± standard deviation (SD). Statistical analyses were carried out using student’s t-test, and differences were considered statistically significant at *p < 0.05, **p < 0.01, ***p < 0.001.

## Results

### Anti-proliferative effects exerted by HMF on HCT-116 cells

**Fig 1:**
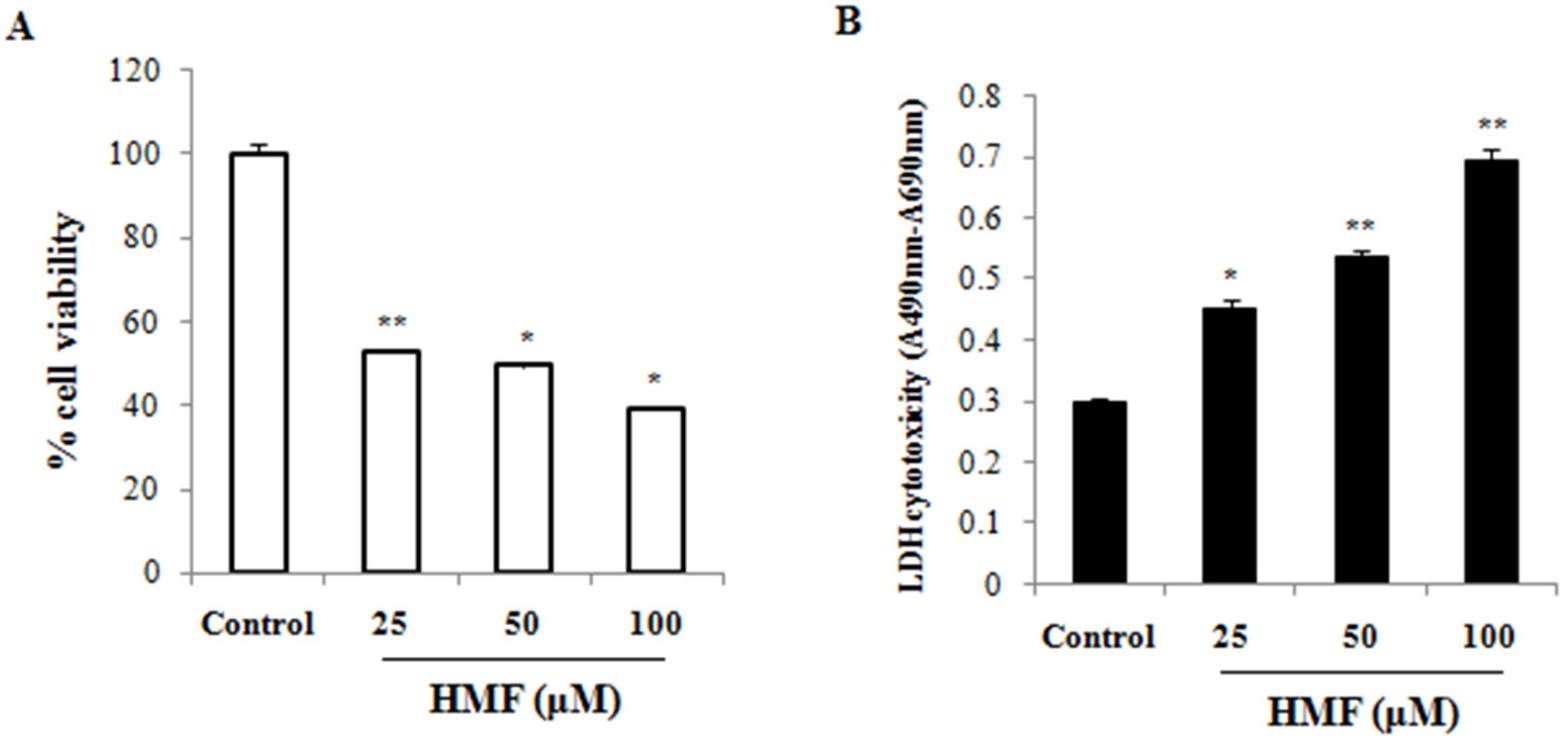
Concentration-dependent effect of HMF on colorectal cancer cell viability. Cells were incubated with various concentrations of HMF for 24 h, after which cell viability was measured using (A) MTT and (B) LDH assays. MTT assay demonstrated fewer viable HCT-116 cells than normal cells at all concentrations. Data represent the mean ± SD (n = 3) from three independent experiments. Asterisk indicates *p<0.05; **p<0.01.

To investigate the cytotoxic effect of HMF on HCT-116 cells, MTT assay was performed as a measure of cell viability. Active mitochondria in living cells can cleave MTT to form formazan, and the amount produced is directly related to the number of living cells. Cultures of HCT-116 cells were treated with 25, 50, and 100 μM HMF for 24 h. The toxic effect of HMF was dose-dependent, and cell viability was lowest at the highest concentration of HMF (100 μM; 39.06±0.03) (Fig 1A). To further determine the lethal effect of HMF exposure for 24 h on HCT-116 cells, LDH assay was performed. LDH is an intracellular enzyme and common marker of cellular integrity, as LDH activity from cultured cells is derived from dead cells only. The results show that LDH activity significantly increased upon HMF treatment in a concentration-dependent manner compared to the control (Fig 1B). Further, viable cell growth was significantly suppressed upon treatment with 25 μM HMF, and the strongest growth reduction was observed in 100 μM HMF-treated cells. The anti-proliferative effect of HMF determined by LDH assay was similar to that determined by MTT assay. Similar concentrations of HMF treatment for 24 h were non-toxic in mouse macrophage Raw 264.7 cells (data not shown). These results show that HCT-116 cancer cells were more vulnerable to HMF than Raw 264.7 cells.

### Significant chromatin fragmentation induced by HMF in HCT-116 cells

**Fig 2:**
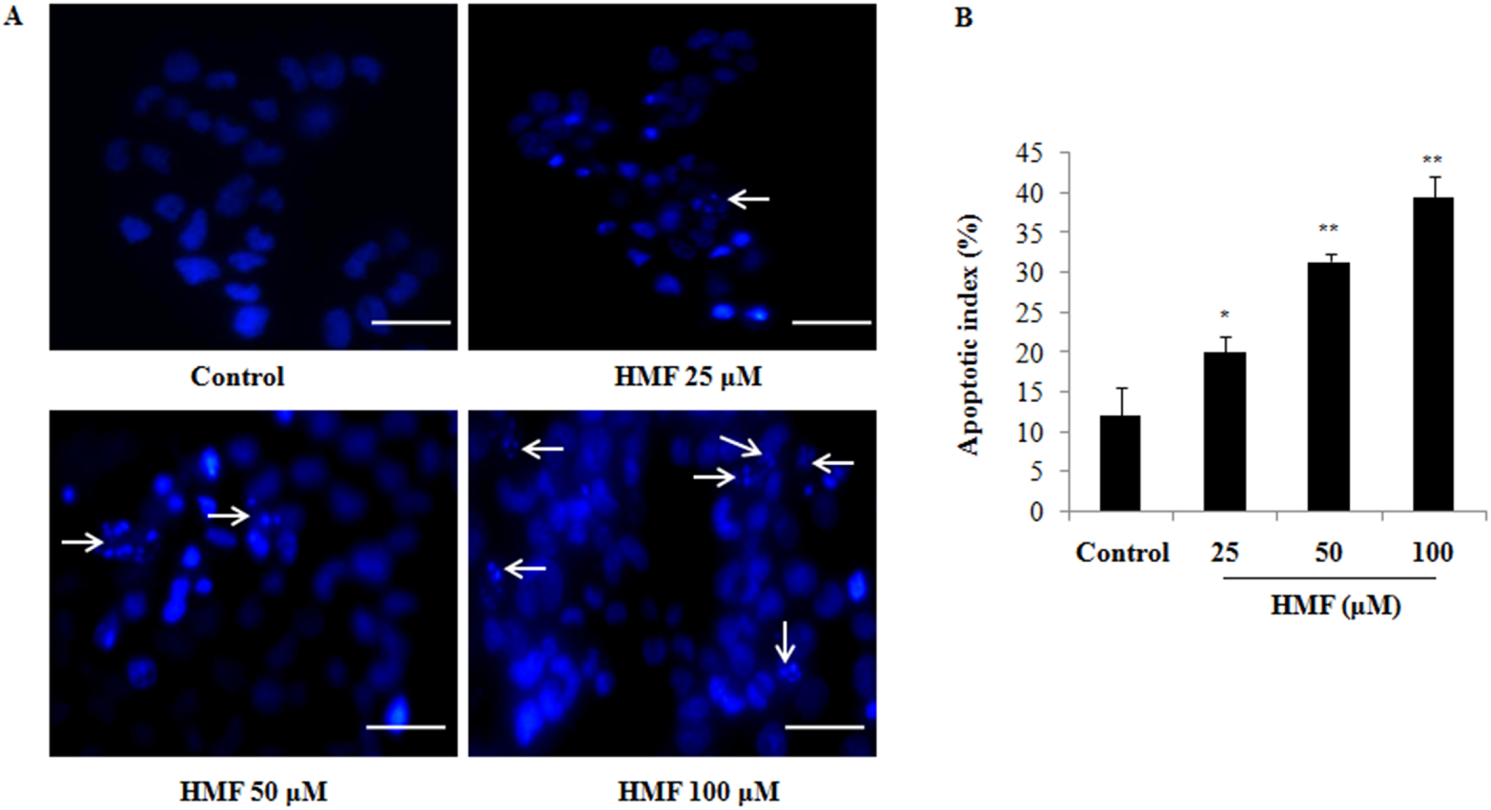
Induction of colon carcinoma cell apoptosis by HMF. (A) After 24 h of incubation, HCT-116 cells were stained with Hoechst 33342, after which cell nuclei were observed under a microscope for detection of apoptosis. The number of apoptotic cells (strong blue staining) significantly increased compared to the control group in a dose-dependent manner. (B) Apoptotic index of HMF-treated cells was significantly higher than the control group. Apoptotic index was calculated as the percentage of apoptotic nuclei compared to the total number of cells and is presented as the mean ± SD (n = 10). Data represent the mean ± SD (n = 3) from three independent experiments.*p<0.05; **p<0.01. Scale bars 0.1 mm.

Apoptosis is a prominent route of cell inactivation characterized by DNA damage induction. The pro-apoptotic effect of HMF on nuclear fragmentation and chromatin condensation in HCT-116 cells was quantified using Hoechst 33342 stain assay. Cells were treated with 25, 50, and 100 μM HMF for 24 h and analyzed. Apoptotic cells were examined under a fluorescence microscope (Fig 2A). Cells with brightly colored, condensed, or fragmented nuclei were considered as apoptotic. The number of cells showing apoptotic morphology was counted in randomly selected fields per well. Differences in chromatin condensation and apoptotic index of HCT-116 cells treated with different concentrations of HMF were significant, indicating that DNA damage was involved in HMF-induced apoptosis in colon cancer cells (Fig 2A and 2B).

### ROS production and oxidative stress induced by HMF in HCT-116 cells

**Fig 3:**
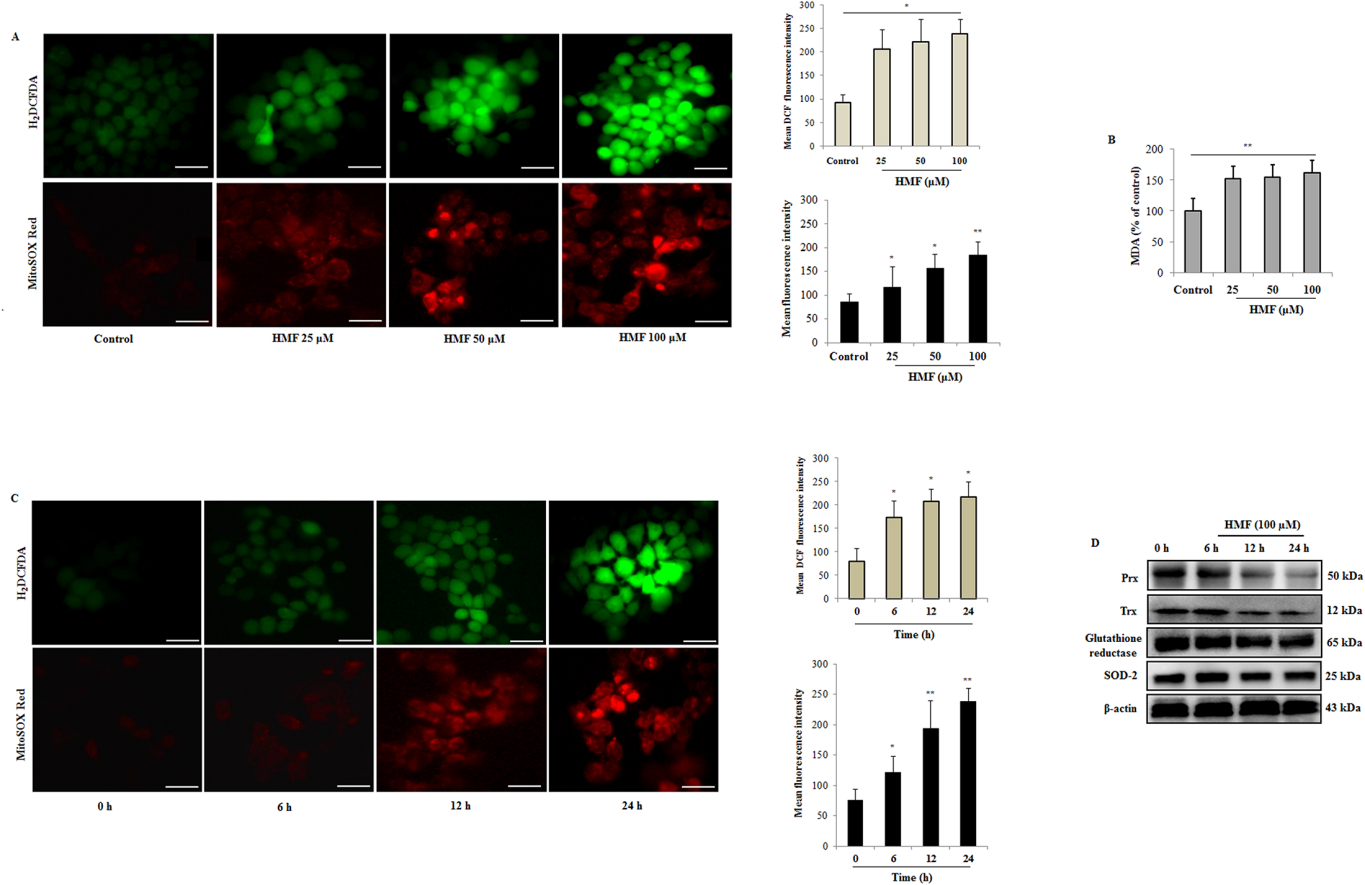
HMF significantly induces ROS generation in HCT-116 cells. (A) Cytosolic and mitochondrial ROS level was measured using the H_2_DCFDA and MitoSOX red fluorescence probe method respectively as described in materials and methods. Mean fluorescent intensity of ROS was analyzed by ImageJ software and presented as the mean ± SD (n = 10). Fluorescence intensity significantly increased in HMF-treated groups. (B) MDA levels were examined by the TBA method. MDA levels were significantly elevated in HMF-treated groups. (C) Cytosolic and mitochondrial ROS generation in time-dependent manner was measured using the H_2_DCFDA and MitoSOX red fluorescence probe methods, respectively. Fluorescence intensity significantly increased in time course manner by HMF treatment. (D) Antioxidant marker enzymes including Prx, Trx, glutathione reductase and SOD-2 was analyzed by western blotting. β-actin was utilized as a loading control. Data represent the mean ± SD (n = 3) from three independent experiments.*p<0.05; **p<0.01. Scale bars 0.1 mm.

We explored the underlying mechanisms leading to HMF-induced cancer cell death. Several studies have previously reported that ROS are mediators of intracellular signaling cascades and can trigger a series of programmed cell death pathways. Here, we used H_2_DCFDA as an intracellular probe to examine cellular ROS levels. Mean DCF fluorescence intensity showed a significant and dose-dependent increase in human HCT-116 cells treated with HMF for 24 h, which means an increase in intracellular ROS levels (Fig 3A). Next, we measured mitochondrial ROS production under HMF treatment using MitoSOX Red, a fluorogenic superoxide detecting dye that specifically targets mitochondria. HMF treatments also effectively elevated mitochondrial superoxide anion generation in dose-dependent manner (Fig 3A). Additionally, we measured MDA levels, which is an oxidative product and an important marker of pro-oxidative status. Our results show that MDA activity increased dose-dependently after HMF treatment compared to untreated cells (Fig 3B). Taken together, these data suggest that HMF acted as a pro-oxidant and had a marked oxidative impact on cellular redox status, leading to induction of cell death. A similar effect was also detected by HMF treatment in time course manner that causes significant increase in cytosolic and mitochondrial ROS production (Fig 3C). Additionally, the expression levels of antioxidant enzymes were examined upon HMF treatment in a time course manner. Western blot analysis revealed reduced expression of thioredoxin (Trx) which induces reduction of oxidized proteins, and peroxire-doxin (Prx) that exhibits Trx-dependent peroxidase activity from 6–24 h during HMF treatment (Fig 3D). Moreover, expression of enzyme glutathione reductase (GR) which catalyzes the reduction of GSSG to GSH, was found to be lowered in time dependent manner (Fig 3D). Mitochondrial tetrameric MnSOD (SOD-2) is pivotal antioxidant enzyme for cell survival which scavenges superoxide anions, and converts them to hydrogen peroxide and oxygen [25]. Our data revealed that HMF treatment reduced the expression levels of SOD-2 in time dependent manner (Fig 3D). These indicate that HMF results in decline of antioxidant defense mechanism which further galvanized ROS production in HCT-116 cells.

### HMF disrupts mitochondrial membrane potential and induces apoptosis

**Fig 4:**
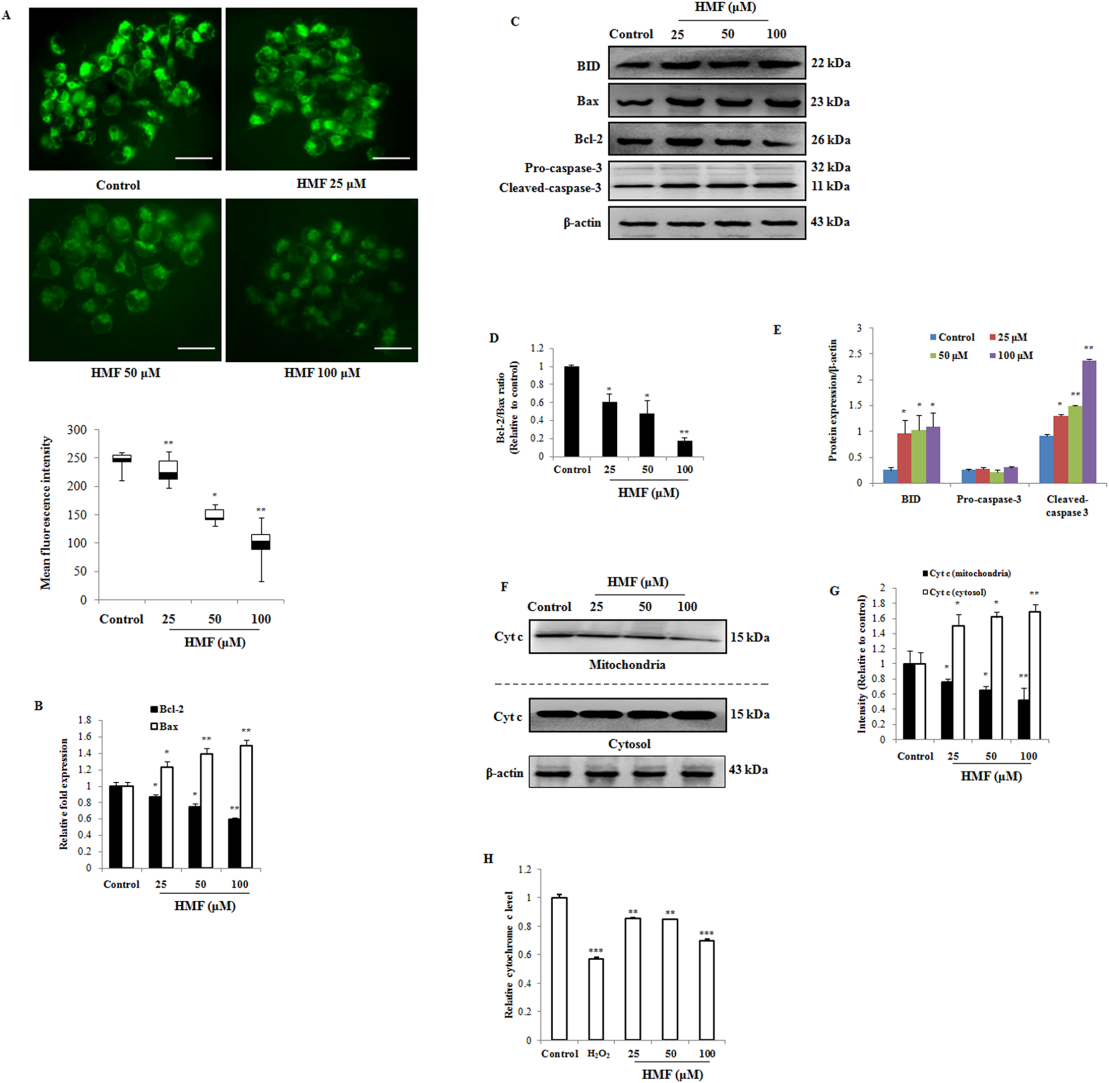
Effect of HMF on disruption of mitochondrial membrane potential and Cyt c release in HCT-116 cells. (A) Cells were treated with the indicated concentrations of HMF for 24 h and stained with Rhodamine-123. Mean fluorescence intensity was quantified using ImageJ software and presented as the mean ± SD (n = 10). (B) Expression profile of *bax* and *bcl-2* gene was assayed using real-time quantitative RT-PCR. Data represent fold changes versus control cells. Data were normalized to housekeeping genes, *β-actin* and *b2m*. (C) Markers of apoptosis, including BID, Bax, Bcl-2, and Caspase-3, were detected by Western blotting. (D) Alteration of Bcl-2 and Bax in HCT-116 cells after 25, 50, and 100 μM HMF treatment for 24 h. (E) Results of Western blotting were analyzed by ImageJ software. (F) and (G) Cyt c release from mitochondria into cytosol in HCT-116 cells after 25, 50, and 100 μM HMF treatment for 24 h was detected by Western blotting. (H) Release of Cyt c from mitochondria in HCT-116 cells was analyzed by measuring the absorbance at 550 nm. Data represent the mean ± SD (n = 3) from three independent experiments.*p<0.05; **p<0.01. Scale bars 0.1 mm.

Elevated intracellular ROS levels may be an indication of mitochondrial dysfunction. In order to examine the effect of HMF on apoptosis induction, loss of MMP was determined by using Rh-123 fluorescent stain. Uptake of Rh-123 by intact, energized mitochondria is positively correlated with MMP, whereas reduction of green Rh-123 fluorescence reflects collapse of MMP (Δψm) and mitochondrial dysfunction in dead cells. As shown in Fig 4A, treatment of cells with various concentrations of HMF led to loss of MMP. Mean fluorescence intensity of Rh-123 was significantly reduced upon treatment with 50 and 100 μM HMF compared to the control.

Furthermore, quantitative RT-PCR and Western blotting results show that the Bcl-2/Bax ratio, which reflects maintenance of mitochondrial membrane stability and susceptibility of cells to apoptosis, significantly decreased in a dose-dependent manner upon HMF treatment (Fig 4B–4D). This result suggests that HMF regulated the expression of Bax and Bcl-2 at both transcriptional and translational levels. Release of Cyt c from mitochondria triggers activation of caspases and apoptotic cell death. In our results, HMF up-regulated cytosolic Cyt c and reduced mitochondrial Cyt c levels in a dose-dependent manner compared to the control group (Fig 4F–4I). Additionally, expression level of pro-apoptotic marker BID increased dose-dependently compared to the untreated group (Fig 4C and 4E). Since caspase activation is a key event in apoptosis, we assessed the effect of HMF on caspase-3 activation in HCT-116 cells. Cleavage of effector caspase-3 in HMF-treated cells dose-dependently increased compared to control cells (Fig 4C and 4E). These results suggest that HMF induced death of HCT-116 cells by regulating the Bcl-2/Bax ratio to activate the intrinsic apoptosis pathway.

### HMF causes ER stress induction and elevates intracellular free calcium levels

**Fig 5:**
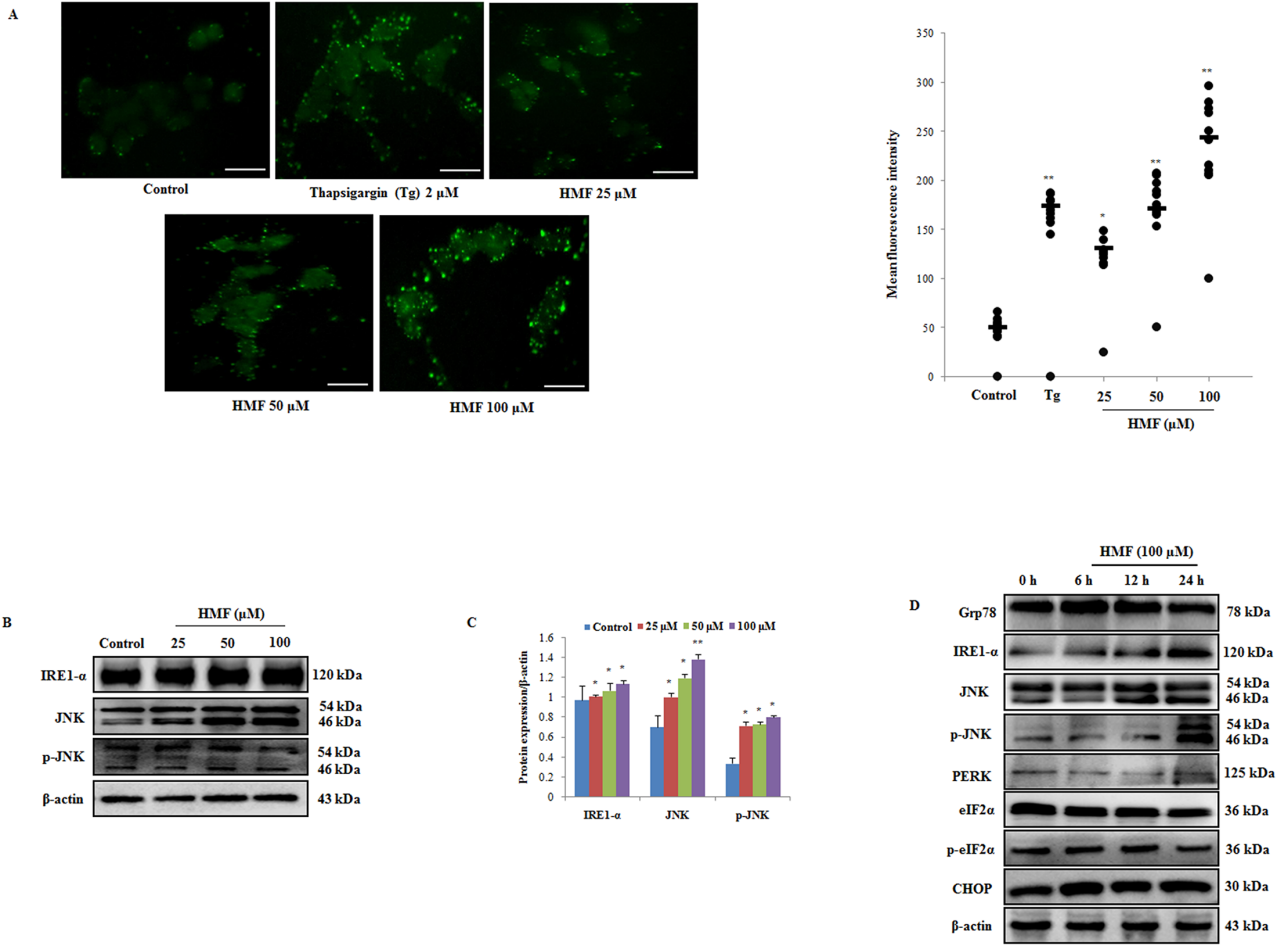
HMF-induced increase in cytosolic Ca^2+^and ER stress activation. (A) Cells were loaded with fura-2 AM, and intracellular calcium release was observed after HMF treatment for 24 h. Thapsigargin (Tg) was used as a positive control. Positive rate of fura-2AM staining was analyzed by ImageJ software and presented as the mean ± SD (n = 10). (B) Western blot analysis of ER stress markers was performed using antibodies specific for IRE1-α, JNK, and p-JNK. β-actin was utilized as a loading control. (C) Densitometry analysis of respective proteins was carried out using ImageJ software, and results were normalized with β-actin in respective controls. (D) Determination of ER stress markers by HMF treatment in a time course manner was determined by western blotting. β-actin was utilized as a loading control. Data represent the mean ± SD (n = 3) from three independent experiments.*p<0.05; **p<0.01. Scale bars 0.1 mm.

Earlier reports have suggested that various anti-cancer drugs are involved in activation of the ER stress-induced c-Jun N-terminal kinase (JNK) pathway, which is a common phenomenon in stress-induced apoptosis. To determine whether or not ROS induction might lead to activation of the JNK signaling pathway, we measured changes in intracellular calcium levels after HMF treatment by using Fura 2-AM stain. When using this stain, an increase in green fluorescent dots is positively correlated with enhanced intracellular calcium levels. As shown in Fig 5A, HMF treatment resulted in more positively stained cells compared to the control, indicating intracellular Ca^2+^ overload. Intracellular Ca^2+^ levels were significantly elevated by HMF treatment in a dose-dependent manner and reached a peak at 100 μM HMF. Thapsigargin (Tg), a sesquiterpene alkaloid is selective inhibitor of SERCA (sarco-/ endoplasmic reticulum calcium ATPase), which induces ER stress by blocking calcium pumping into ER and reduces ER luminal Ca^2+^ concentrations [26]. Tg treatment causes increase in intracellular Ca^2+^ levels which initiates ER stress (Fig 5A).

Regarding cell death, mitochondria and the ER seem to be physically and physiologically coupled [27]. During apoptosis, Ca^2+^ release from ER stores mediated by inositol triphosphate receptors (IP_3_Rs) has been implicated for calcium loading in mitochondria [28]. This calcium overloading mechanism in mitochondria is paralleled by release of pro-apoptotic proteins [29]. It is well established that intracellular Ca^2+^ overload has a toxic effect on cells, resulting in activation of cellular death mechanisms [30]. As the ER plays a crucial role in maintaining intracellular Ca^2+^ homeostasis, HMF treatment led to disruption of Ca^2+^ levels and triggered ER stress. We therefore assessed protein expression levels of the ER stress transmembrane protein IRE1-α. Upon exposure of HCT-116 cells to various concentrations of HMF, expression levels of IRE1-α increased in dose-dependent manner and reached a peak at 100 μM HMF. Furthermore, Fig 5B and 5C shows that HMF induced concentration-dependent phosphorylation of JNK in parallel with increased protein expression of IRE1-α, suggesting that JNK was activated in response to HMF-induced ER stress. JNK activation is known to cause phosphorylation of Bcl-2, resulting in apoptosis via enhanced expression of pro-apoptotic protein Bax and inhibited expression of Bcl-2. As previously explained in Fig 4B and 4C, HMF-mediated up-regulation of Bax may be caused by JNK phosphorylation, resulting in apoptosis activation. Additionally, we also checked the effect of HMF on expression ER stress markers in time dependent manner. Glucose regulated protein (Grp78) is a major ER chaperone protein which binds to UPR transducers and releases them upon encountering ER stress [31]. Our results show that HMF treatment after 6 h, slightly increases Grp78 expression but downregulates at 12 and 24 h treatment, which results in increase in IRE1-α expression and JNK activation Furthermore, we check the effect of HMF treatment on the expression levels of PERK- eIF2α-CHOP pathway, which is responsible for ER stress mediated apoptosis [32]. However, no change in the expression level of PERK was detected, and expression of eukaryotic initiation factor2-alpha (eIF2α), phospho-(eIF2α) and C/EBP-homologous protein (CHOP) were found to be reduced. These results further suggest that HMF mediated apoptosis induction is mediated by downregulating Grp 78 expression, which activates IRE1-α-JNK pathway and is independent of PERK- eIF2α-CHOP pathway (Fig 5D).

### NAC mitigates mitochondrial-mediated apoptosis via ROS removal

**Fig 6:**
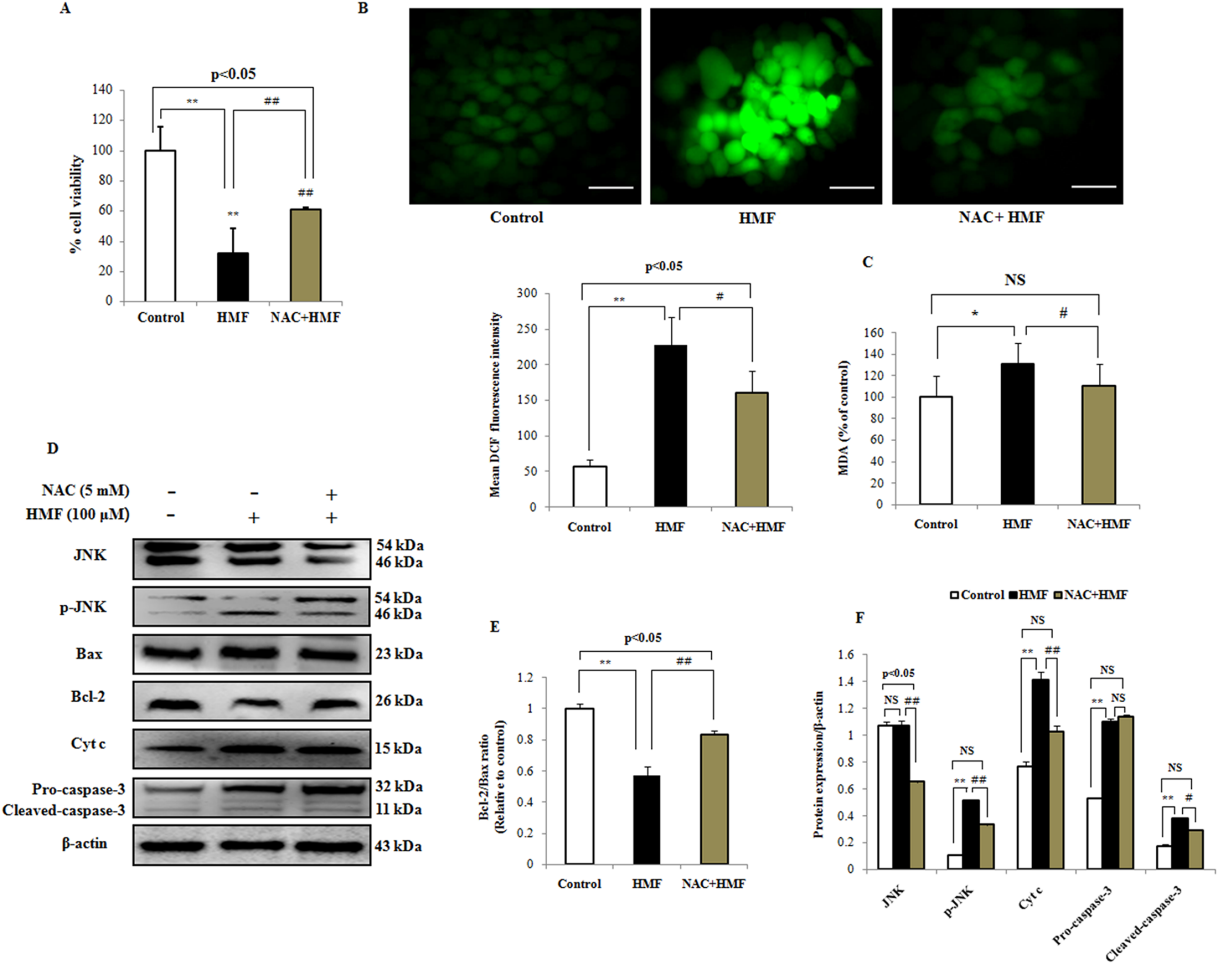
Role of ROS in apoptosis induced by HMF. HCT-116 cells were pre-incubated with NAC (5 mM) for 12 h, followed by treatment with HMF (100 μM) for 24 h. (A) Cell viability was measured by MTT assay. (B) Level of ROS generation was measured by H_2_DCFDA staining. Quantitative analysis of ROS generation was carried out using ImageJ software and shown in histograms. Data are presented as the mean ± SD (n = 10). (C) Treatment with 5 mM exogenous NAC significantly reduced MDA levels. (D) and (E) NAC reduced phosphorylation of JNK, resulting in attenuation of Bax and Cyt c expression, inactivation of caspase-3, and reduction of apoptosis. (F) Densitometry analysis of respective proteins was carried out using ImageJ software, and results were normalized with β-actin with respect to controls. Data represent the mean ± SD (n = 3) from three independent experiments.*p<0.05; **p<0.01 vs control, #p<0.05; ##p<0.01 vs HMF treatment. NS indicates not significant. Scale bars 0.1 mm.

To confirm the role of ROS in HMF-induced cell death, the ROS scavenger NAC was used. For this, we co-incubated HCT-116 cells with 5 mM exogenous NAC for 12 h and 100 μM HMF for 24 h. After co-incubation of NAC with HMF, MTT assay showed that NAC protected against cell death (Fig 6A). In detail, Fig 6B shows that NAC blocked the ability of HMF to stimulate ROS induction, and mean fluorescence intensity was reduced. NAC also significantly reversed elevation of MDA levels caused by HMF treatment (Fig 6C). NAC directly inhibited HMF-induced mitochondrial dysfunction as well as potently attenuated JNK phosphorylation and apoptosis (Fig 6D and 6F). Further, reduction of Bcl-2 expression by HMF was prevented, whereas expression levels of Bax, Cyt c, and cleaved caspase-3 were reduced (Fig 6D–6F). These results reveal that ROS act as proximal mediators for initiation of HMF-induced mitochondrial apoptosis.

## Discussion

**Fig 7:**
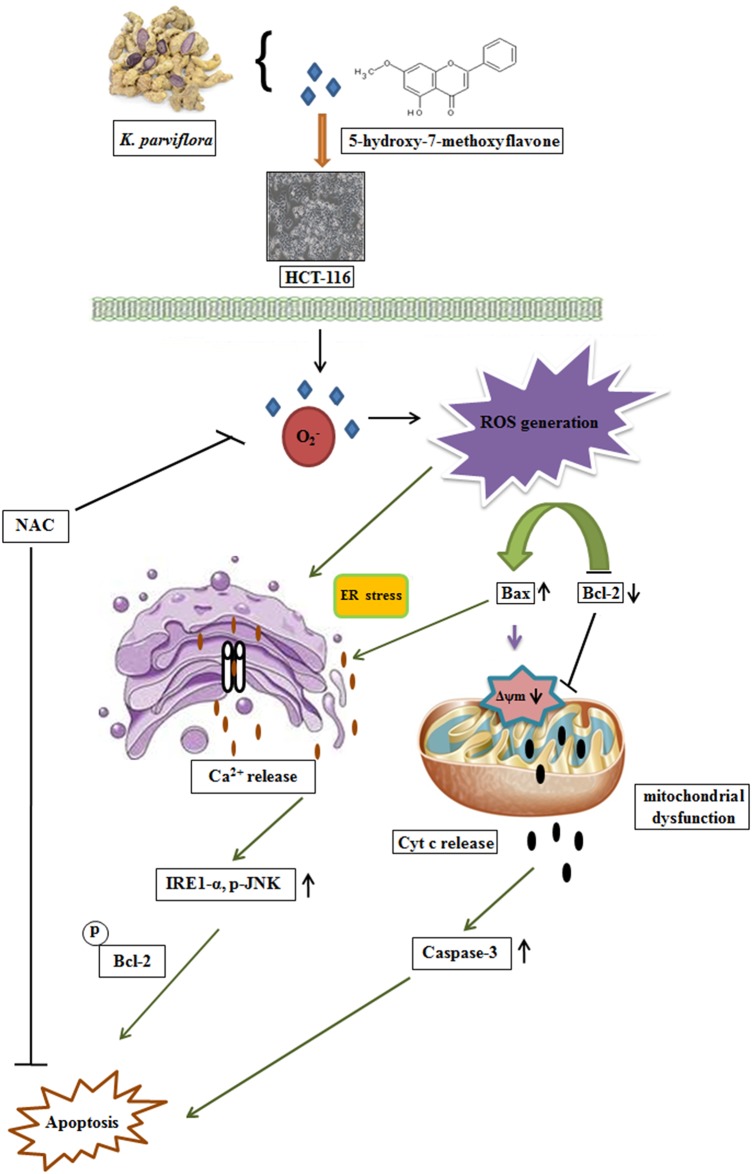
Schematic representation of plausible detailed molecular mechanism of HMF-induced cell death by ROS-mediated intrinsic apoptosis pathway. HMF treatment leads to ROS generation and Ca^2+^ release, resulting in ER stress induction. Simultaneously, HMF causes alteration of mitochondrial membrane potential (MMP) and reduction of the Bcl-2/Bax ratio, leading to activation of caspase-3 and apoptosis progression. In contrast, ROS inhibition by NAC attenuates HMF-induced mitochondrial apoptosis in HCT-116 cells.

Apoptosis induction is a widely accepted approach to cancer treatment, as most cancer cells exhibit abnormal regulation of apoptosis. Natural compounds that specifically target colon cancer cells but are less toxic to normal colonic epithelial cells have potential for colorectal cancer treatment [33]. Our results presented here confirm that ROS are critical modulators of HMF-mediated apoptosis in human colon cancer *in vitro*. This novel finding is verified by the following evidence: (i) HMF dose-dependently inhibited proliferation of colon carcinoma cells and promoted chromatin condensation. (ii) HMF-triggered ROS accumulation was accompanied by ER stress, resulting in activation of the IRE1-α-JNK pathway. (iii) HMF-induced cell death was due to alterations in Δψm, Cyt c release, and activation of apoptogenic proteins. (iv) ROS inhibition reversed the effects of HMF and promoted cell survival by blocking JNK phosphorylation and attenuation of apoptosis (Fig 7).

In the present study, HMF induced suppression of colon cancer cell growth in a dose-dependent manner (Fig 1). It has been suggested that the hydroxyl group at the 5-position of PMF plays a pivotal role in inhibition of cancer cell proliferation [16]. This may be associated with the relative lipophilicity of the compound, as it promotes binding to the cellular plasma membrane, resulting in enhanced uptake into the cytosol [16]. Additionally, methylated flavones with a methoxy group at the 7-position show high hepatic metabolic resistance and have high rates of intestinal absorption compared to unmethylated analogs. Thus, methylated flavones are orally bioavailable and have great potential as chemotherapeutic agents [34].

The most well-known route of cancer eradication is by induction of apoptosis, which plays a key role in the pathogenesis of cancers. Thus, targets related to apoptosis are of prime interest in cancer therapy. Accumulating evidence suggests that flavonoid-induced apoptosis is characterized by nuclear condensation, disruption of mitochondrial membrane integrity, and up-regulation of apoptogenic proteins [35, 36]. Here, our results demonstrate that HMF induced the intrinsic apoptosis pathway. HMF-treated cells visualized using Hoechst 33342 stain exhibited an increase in nuclear chromatin staining characterized by morphological features of apoptosis, including increased nuclear shrinkage and pycnonuclei formation (Fig 2). Despite being the ‘powerhouse’ of cells under aerobic conditions, mitochondria can also be the source of signals that promote apoptotic insults. Reduction of mitochondrial membrane potential (Δψm) due to depolarization of the mitochondrial membrane, increased membrane permeability due to perforation of Bax, release of Cyt c into the cytoplasm, translocation of BID from mitochondria, and proteolytic caspase cascade activation have all been reported in response to apoptotic stimuli [37, 38]. Our findings show that HMF treatment depleted Δψm, stimulated release of Cyt c into the cytosol, and significantly increased BID and caspase-3 levels (Fig 4). Further, the Bcl-2/Bax ratio, which represents mitochondrial function, was altered by HMF treatment. Existing evidence supports that natural compounds such as curcumin, cordycepin, and others mediate their anti-carcinogenic effects by activating intrinsic apoptosis [39–41].

Elevation of free intracellular Ca^2+^ levels is mediated by the ER. Since the ER and mitochondria are in close vicinity within cells, ER-derived Ca^2+^ trafficking ER can trigger a variety of signaling mechanisms promoting Ca^2+^-mediated mitochondrial death [27]. Activation of the UPR pathway is a response mechanism to stress stimuli. In this study, intracellular Ca^2+^ release increased in a dose-dependent manner in response to HMF treatment (Fig 5A), leading to activation of the IRE1-JNK pathway (Fig 5B). Further, HMF treatment led to significant up-regulation of IRE1-α expression and JNK phosphorylation (Fig 5B and 5C). Previous studies have shown that the JNK signaling pathway is activated in response to changes in calcium flux, resulting in recruitment of TRAF2 by IRE1 to form the TRAF2-ASK1-IRE1 complex [42]. Our results are in agreement with previous studies in which JNK was shown to be activated in response to docetaxel-induced ER stress via the IRE1-α pathway in human melanoma cells [43]. Earlier reports have shown that Bak and Bax are involved in Ca^2+^-mediated apoptosis, as overexpression of Bax was found to be required for ER-derived Ca^2+^ release with a concomitant increase in mitochondrial Ca^2+^ and Cyt c release [44]. In addition to its role in mitochondrial apoptosis, anti-apoptotic Bcl-2 has a cytoprotective effect against ER stress. It is believed that JNK-mediated phosphorylation of Bcl-2 can inhibit anti-apoptotic properties, leading to increased Ca^2+^ release from the ER, mitochondrial Ca^2+^ uptake, and apoptosis [45]. Grp78, which maintains the inactive state of IRE1- α and PERK in unstressed conditions, disjoins and activates these enzymes during ER stress, was reduced by HMF treatment. Grp78 is involved in various cellular processes including, maintaining Ca^2+^ homeostasis and as central regulator of UPR [31]. Earlier reports suggested that knockdown of Grp78 results in increased phosphorylation of JNK, a downstream target of IRE1- α [31]. Our results suggest that HMF treatment causes downregulation of Grp78 and upregulates IRE1- α and JNK expression levels in time course manner (Fig 5D). Furthermore, no change in the expression levels of PERK, p-eIF2α and CHOP proteins (Fig 5D) suggest that HMF mediated cell death is independent of PERK- eIF2α-CHOP pathway. Therefore, it can be concluded that HMF causes up-regulation of intracellular Ca^2+^, which downregulates Grp78 expression leading to activation of the IRE1-α-JNK pathway and apoptosis.

It is noteworthy that ROS are mediators of intracellular signaling cascades and can trigger mitochondria-associated damage events such as apoptosis, which deviates from the “canonical” view of malignant progression of cancer cells [46, 47]. However, the identities of the cellular sources of ROS generation and apoptosis induction in various cell types remain controversial. Therefore, intracellular ROS can be exploited for cancer therapy either by boosting ROS production with drugs or by diminishing ROS to obstruct early tumorigenesis. Phytochemicals such as polyphenols are considered as “double-edged swords” in the cellular redox state since they exhibit dual behavior *in vitro*. The precise mechanism by which HMF cause increased levels of mitochondrial ROS remains undetermined. The reasons are manifold. We speculate that HMF may diffuse through the cell membrane into the cytoplasm and have a cytotoxic effect by generating ROS, resulting in oxidative stress in HCT-116 cells. HMF treatment may induce mitochondrial dysfunction which augments mitochondrial ROS generation in time dependent manner (Fig 3A and 3C). Furthermore, HMF treatment provoked decreased antioxidant enzymes expression which further enhances ROS generation (Fig 3D). Moreover, earlier reports have suggested that an increase in [Ca^2+^]_i_, potentiates ROS production from mitochondrial and cytosolic sources [48–50]. In addition, ROS may interfere with Ca^2+^ adenosine triphosphatases (ATPases) and other mechanism that regulate intracellular calcium homeostasis, suggesting that calcium and ROS can act concomitantly to affect cell survival [51]. It has been well documented that natural compounds exhibiting anti-cancer properties are potent ROS generators and thus could interfere with cellular signaling cascades [52, 53]. In the present study, HMF led to increased cytosolic and mitochondrial ROS generation, MDA levels and decreased antioxidant expression levels (Fig 3). The ROS inhibitor NAC strongly blocked ROS generation, diminished MDA levels, and abolished JNK phosphorylation (Fig 6B–6D). Attenuation of pro-apoptotic markers was observed while cell viability was enhanced as assessed by MTT assay (Fig 6D, 6E, 6F and 6A). Based on these data, we can conclude that HMF induces apoptosis through ROS generation, which plays a vital role in the intrinsic apoptosis pathway.

## Conclusion

Our study is the first to demonstrate that HMF can effectively induce cell death of colorectal cancer cells by the ROS-mediated mitochondrial apoptosis pathway. These compelling results expand our understanding and may be helpful for the development of HMF into a chemotherapeutic drug for treatment of colorectal cancer.
